# A follow-up study of 100 patients with acute brucellosis for its prognosis and prevention

**DOI:** 10.3389/fmed.2023.1110907

**Published:** 2023-11-03

**Authors:** Long Bai, Na Ta, Aoning Zhao, Huhe Muren, Xueyong Li, Buhe Chaolu Wang, Hurile Bagen, Yongjun Wen

**Affiliations:** ^1^Inner Mongolia Medical University College of Mongolian Medicine and Pharmacy, Hohhot, China; ^2^Inner Mongolia Agricultural University, Hohhot, China; ^3^Inner Mongolia Center for Disease Control and Research, Hohhot, China; ^4^Vanke School of Public Health, Tsinghua University, Beijing, China; ^5^Chi Feng Center for Disease Control and Research, Chifeng, China

**Keywords:** brucellosis, case follow-up, repeated measures, antibody titres, biochemical parameters, clinical symptoms

## Abstract

**Objective:**

To prevent chronic brucellosis, this study analysed the changes in patient antibody titers, and the trajectories of biochemical indicators at different stages of brucellosis, identified relevant biomarkers, and explored risk factors affecting the prognosis of brucellosis patients.

**Methods:**

A prospective cohort study was conducted to follow 100 patients with acute brucellosis. Laboratory serological test results [taken with a serum (tube) agglutination test (SAT)] and biochemical parameters (liver function, renal function, and hematological system) were measured repeatedly at four-time points: 0 weeks—baseline survey, 6 weeks after the first treatment, 12 weeks after the second treatment, and 3 months after the third treatment. The changes in the antibody titres and biochemical parameters at each time point were analysed for trend changes.

**Results:**

One hundred patients with acute brucellosis were enrolled in this follow-up study, with 100% retention in follow-up. By the third follow-up, 21 patients had turned subacute and 11 had turned chronic. One-way repeated measures analysis of variance results showed statistically significant differences (*p* < 0.01) across the time points for the following five indicators: alanine aminotransferase, aspartate aminotransferase, total bilirubin, serum creatinine (SCr) and platelet count. The clinical symptoms of patients in the acute stage were mainly joint pain, fatigue, and fever, while those in the chronic stage complained primarily of joint pain and fatigue. The results of multivariate logistic analysis showed that joint pain [odds ratio (OR) = 3.652, 95% confidence interval (CI) =1.379–9.672], monoarticular pain (OR = 6.356, 95% CI = 4.660–8.669), elevated SCr (OR = 15.804, 95% CI = 1.644–151.966) and elevated haemoglobin (Hb) (OR = 1.219, 95% CI = 1.065–1.736) were risk factors for poor prognosis (not cured or chronic) in patients with brucellosis.

**Conclusion:**

The trajectory of changes in patient SAT posirates and antibody titers can be used to distinguish patients with chronic brucellosis. The brucellosis is preventable and treatable, and the standard treatment can be effective in reducing the clinical symptoms of affected patients. If patients are not treated in a timely manner, joint pain, monoarticular pain, and elevated SCr are risk factors for patients who are not cured. Therefore, the treatment cycle for these patients should be extended.

## Highlights

- Here is what is already known on this topic:

1. Brucellosis is a zoonotic allergic disease caused by bacteria of the genus *Brucella* and has become an important public health problem worldwide.
2. Common symptoms after *Brucella* infection in humans include fever, fatigue, loss of appetite and joint pain.
3. Chronic brucellosis has atypical clinical manifestations that are often recurrent and clinically difficult to cure.

- Here is what this study adds:

1. The early diagnosis and standard treatment of patients with acute brucellosis can effectively control the disease.
2. The serum (tube) agglutination test positive rate and antibody titre gradually decrease over the course of the acute phase of brucellosis.
3. After receiving uniform standard treatment, patients’ symptoms (e.g., fever, fatigue, hyperhidrosis and polyarticular pain) decreased continuously.
4. Joint pain and single joint pain are risk factors for patients’ failure to be cured.

- Here is how this study might affect research, practice or policy:

1. There is a certain trajectory between the course of acute brucellosis and the serum (tube) agglutination test positive rate, antibody titre and alanine aminotransferase level.
2. Patients in the acute phase of brucellosis can effectively relieve and eliminate their clinical symptoms through a full course of standardised treatment.
3. The treatment cycle should be extended for patients who develop certain symptoms such as joint pain.

## 1. Introduction

Brucellosis is a zoonotic disease caused by bacteria of the genus *Brucella* (1) and a typical natural epidemic infectious disease. Common symptoms of brucellosis in humans include fever, malaise, loss of appetite, and joint pain, which in turn may lead to complications such as meningitis and arthritis, affecting the patient’s ability to work and possibly leading to death in severe cases (2). Human-to-human transmission of *Brucella* is rare, while some livestock-related human infections are caused by contact with infected animals such as sheep and cattle, especially during the delivery of lambs (3). *Brucella* can spread via airborne mechanisms in certain circumstances (4, 5). At present, the epidemic situation of brucellosis is seriously underestimated and has become an important global public health concern (6, 7); additionally, the World Health Organization believes that brucellosis is one of the most easily overlooked zoonoses (8).

The clinical symptoms of brucellosis in the acute phase are typical, and can be cured with a full course of timely standardised treatment. Chronic brucellosis, which has atypical clinical manifestations is difficult to cure clinically. Patients with chronic brucellosis often have recurrent clinical manifestations; some patients have serious sequelae, while others even lose their ability to work. Due to the lack of specialised treatment for chronic brucellosis, its repeated symptoms, and its prolonged disease course, patients bear a heavy economic burden due to the medical treatment costs. In addition, the infected animals must be treated, adding an even heavier financial burden to patients with chronic brucellosis. At present, there is no exact laboratory differential test for chronic brucellosis, which is mainly determined according to the duration of the disease; the course of the disease in many patients is mainly determined by the patients’ chief complaints (9), resulting in different disease stages in one patient being determined by various doctors. These issues directly affect the standardised treatment and prognosis of patients with brucellosis, and they even affect the accurate implementation of overall prevention and control in a region. It is reported that approximately 5%–15% of patients with brucellosis in China still change from acute to chronic every year (10). This study analyses whether there is a trajectory in the changes of antibody titres and biochemical parameters during each course of the disease to provide a theoretical basis for finding markers associated with chronic brucellosis. At the same time, through analyzing the relevant contents of the questionnaire, this study determines the risk factors that affect the prognosis of patients with brucellosis and provides a scientific basis for preventing the disease from turning chronic, thus improving the survival quality of patients.

## 2. Materials and methods

### 2.1. Study subjects

This was a prospective study. This study selected the Disease Control and Prevention Centre of Wuchuan County, Inner Mongolia Autonomous Region, as the research site, and it included patients with acute brucellosis according to the Diagnostic Criteria for Brucellosis (11). The confirmed patients were treated in designated medical institutions and completed the study’s baseline questionnaire after giving informed consent.

### 2.2. Inclusion criteria

Patients with brucellosis were included using the Diagnostic Criteria for Brucellosis WS269-2019 (11). They were aged 18–70 years, and the study’s survey was administered according to the principle of informed consent until the sample size was met. According to the Diagnostic Criteria for Brucellosis WS269-2019, patients were included who had an epidemiological history and clinical manifestations of the disease and met any of the following conditions: ① the titre of the serum (tube) agglutination test (SAT) was 1:100 or above; ② the titre of the anti-human immunoglobulin test was more than 1:400; and ③ *Brucella* was isolated. The subacute stage of brucellosis was defined if the patient had clinical symptoms related to brucellosis, the course of the disease was within 3–6 months and the laboratory confirmed a serological positive reaction. The chronic stage of brucellosis was defined if the disease lasted more than 6 months, was not cured and had signs related to brucellosis with a serological positive reaction confirmed by the laboratory.

### 2.3. Exclusion criteria

Patients who met any of the following criteria were excluded: ① those infected with human immunodeficiency virus, undergoing chemotherapy, suffering from other immune system diseases (such as lupus erythematosus) or combined with other serious diseases (such as liver failure, severe hepatitis, liver cirrhosis, renal failure, uraemia, proteinuria, multi-drug resistant pulmonary tuberculosis, severe coronary heart disease, chronic obstructive pulmonary disease, and cerebral infarction); ② patients with mental disorders, deafness, and other diseases resulting in poor communication and unable to cooperate with the survey; ③ pregnant women; ④ patients who did not agree to participate in this study; and ④ patients who were allergic to the therapeutic drugs in this study.

### 2.4. Sample size

Based on previous literature (12) and a 1 year treatment follow-up period, it was assumed that the chronicity rate of brucellosis was approximately 12%. In addition, the test validity was set at Power = 0.90, the test level at *ɑ* = 0.05, and the sample size *N* = 85 was calculated by SPSS software. Considering possible dropouts, 100 patients with brucellosis were included in this study. Here is the sample size calculation formula:


$$n=Z_{\alpha }^{2}P\left(1-P\right)/\delta^{2}$$

*P* is the chronicity rate and *δ* is the allowable error.

## 3. Methods

### 3.1. Treatment plan

All included patients were treated in strict accordance with the Diagnostic Criteria for Brucellosis WS269-2019 (11). The patients were treated with doxycycline (200 mg/d, twice a day) and rifampicin (15 mg/kg, once a day) in accordance with the principles of early, combined and sufficient treatment.

### 3.2. Data collection

Standardised questionnaires were used to collect patient information, including general demographic (age, sex, occupation) and epidemiological data (frequency and mode of exposure to animals). Baseline and follow-up surveys and relevant laboratory tests were performed on all patients with brucellosis who met the study’s inclusion criteria. The study was approved by the Ethics Committee of the Centre for Disease Control and Prevention of Inner Mongolia Autonomous Region (approval number: 2022011601).

### 3.3. Baseline survey

The patients were diagnosed according to the Diagnostic Criteria for Brucellosis WS269-2019 (11), and the study subjects were selected according to the inclusion and exclusion criteria. Informed consent forms were signed by the patients included in the study, and the baseline questionnaires were administered to them for uniform and standardised treatment. Blood was collected from the included patients for the SAT, liver function, kidney function, haematological system and other indexes.

### 3.4. Follow-up investigation

A total of three follow-up surveys were conducted on the included patients, and the follow-up times were 6 weeks after treatment, 12 weeks after treatment, and 3 months after treatment. In addition to the surveys, the SAT, liver function, kidney function, haematological system and other indexes were collected at each follow-up visit.

### 3.5. Laboratory testing

#### 3.5.1. SAT

Unknown patient serum was added to a suspension of *Brucella* antigens, and if *Brucella* antibodies were present in the patient’s serum, then antigens and antibodies reacted specifically to form agglutinates that were visible to the naked eye. The tested serum dilutions were 1:50, 1:100, 1:200 and 1:400. The titre was determined by the highest serum dilution that produced 50% (++) agglutination. Haemagglutination above (++) was considered positive in serum diluted to 1:100 and suspicious in serum diluted to 1:50.

#### 3.5.2. Liver function test

According to the instructions of the kit (manufacturer: Jiangsu Maiyuan Biotechnology Co., Ltd.), a colorimetric determination was performed, the standard curve at a wavelength of 600 nm was prepared, and the alanine aminotransferase (ALT), aspartate aminotransferase (AST), and total bilirubin (TBIL) contents were calculated on a standard curve after the blood sample was measured.

#### 3.5.3. Renal function test

Blood urea nitrogen (BUN) and serum creatinine (SCr) were determined by a semi-automatic biochemical analyser (manufacturer: Mindray; model: BA-88A) in the patient’s serum.

#### 3.5.4. Haematological test

ALT, AST, TBIL, BUN, SCr, red blood cell (RBC) count, white blood cell (WBC) count, haemoglobin (Hb), platelet (PLT) count and other indicators were measured on an automatic haemocytometer (manufacturer: Mindray; model: BC-2600).

### 3.6. Analytical and statistical methods

EPIDATA 3.0 was used to establish a database, and the double entry method was used for questionnaire and experimental data entry. The descriptive analysis and statistical inference were performed using SPSS software version 23.0 (IBM SPSS Statistics for Windows, Version 23.0. Armonk, NY: IBM Corp.), and descriptive statistics were reported as absolute numbers and constituent ratios (*n*, %). Pearson’s chi-square test and a two-sided Fisher’s exact test were used to compare enumeration data between two or more groups, respectively, and *p* < 0.05 was considered statistically significant.

One-way repeated measures analysis of variance (ANOVA) was used for biochemical indicators at different survey time points, and a homogeneity test and sphericity test were performed for the data. If *p* > 0.05 for the sphericity test, the data conformed to the sphericity hypothesis, and there was no correlation between the data, so the repeated measures ANOVA was used. If *p* < 0.05, the data did not conform to the sphericity hypothesis, and the Greenhouse–Geisler correction was used.

Potential factors influencing the prognosis of brucellosis were analysed by a univariate logistic regression analysis, and variables that were significant in the univariate analysis (*p* < 0.05) or variables that were considered likely to affect the treatment outcomes were included in the multivariate logistic regression analysis. The criteria for the assignment are listed in Table 1.

**Table 1:** Variable assignment table for logistic regression analysis.

Independent variable	Index category	Assignment criteria
Gender	Male	1
	Female	2
Age	≥45 years	1
	<45 years	2
Asthenia	Yes	1
	No	2
Arthralgia	Yes	1
	No	2
Shoulder pain	Yes	1
	No	2
Knee pain	Yes	1
	No	2
Monoarticular pain	Yes	1
	No	2
Polyarticular pain	Yes	1
	No	2
Joint migration pain	Yes	1
	No	2
On-time medication	Yes	1
	No	2
Alanine aminotransferase increased	Yes	1
	No	2
Aspartate aminotransferase increased	Yes	1
	No	2
Bilirubin increased	Yes	1
	No	2
Blood urea nitrogen increased	Yes	1
	No	2
Creatinine increased	Yes	1
	No	2
Red blood cell increased	Yes	1
	No	2
White blood cell decreased	Yes	1
	No	2
Hemoglobin increased	Yes	1
	No	2
Platelet decreased	Yes	1
	No	2

## 4. Results

### 4.1. Overview of included cases

According to the case diagnostic screening rules, a total of 100 patients with brucellosis were included in the follow-up study, with 100% retention in follow-up. Out of the total, 61 patients were males, and 39 were females, with a male-to-female ratio of 1.56:1 and a mean age of 53.81 ± 0.99 years. Their occupations were dominated by farmers; 92 patients were farmers, 5 patients were catering industry practitioners, 2 patients were herdsmen, and 1 patient was a worker. At the baseline survey, 98 patients had been exposed to animals within the past month, including 78 dogs (79.59%), 72 sheep (73.47%), 69 pigs (70.41%), 48 cattle (48.98%), and 2 others (2.04%); 97 patients (98.98%) had been exposed to sheep, cattle, dogs, and pigs. By the third follow-up, 32 patients had positive SAT results and associated clinical symptoms. Over the course of this study, 21 patients turned subacute, 11 patients turned chronic, and the chronic rate was 11%.

### 4.2. SAT test results

The baseline SAT test was positive in 100 patients, the SAT antibody titre was ≥1:400^++^ in 4 patients, the antibody titre was ≥1:200^++^ in 89 patients and the antibody titre was ≥1:100^++^ in 7 patients.

At the first follow-up visit, there were 82 SAT-positive patients without SAT antibody titres ≥1:400^++^, 76 (92.68%, 76/82) patients with antibody titres ≥1:200^++^ and 6 (7.32%, 6/82) patients with antibody titres ≥1:100^++^.

At the second follow-up visit, there were 58 SAT-positive patients without SAT antibody titres ≥1:400^++^, 50 (86.21%, 50/58) patients with antibody titres ≥1:200^++^ and 8 (13.79%, 8/58) patients with antibody titres ≥1:100^++^.

At the third follow-up visit, there were 32 SAT-positive patients without SAT antibody titres ≥1:400^++^, 18 (56.25%, 18/32) patients with antibody titres ≥1:200^++^ and 14 (43.75%, 14/32) patients with antibody titres ≥1:100^++^.

**Figure 1:**
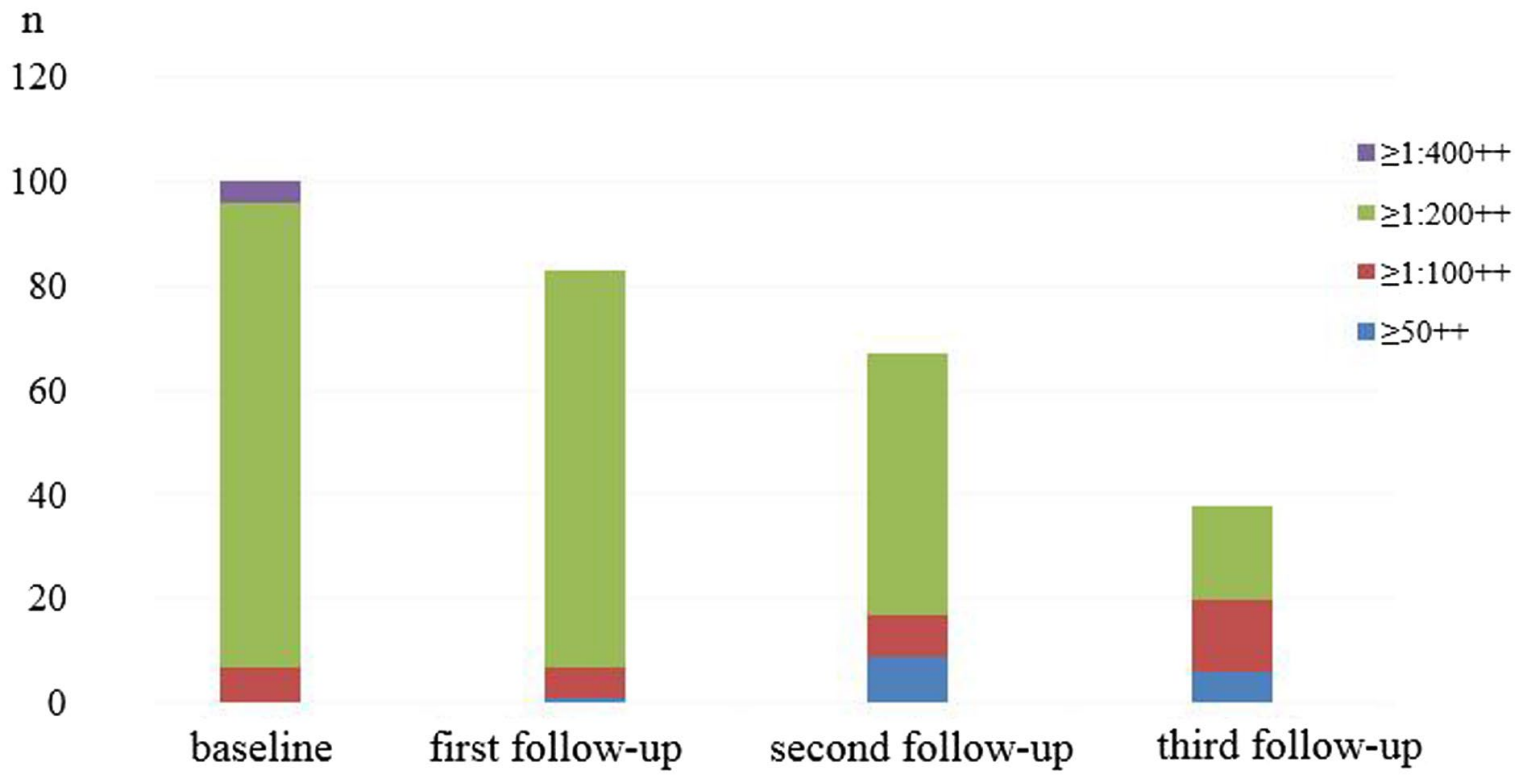
Antibody titer composition of SAT test at each survey time.

The trend of antibody titre composition and the positive rate at each survey time point is shown in Figure 1.

The positive rates of the four survey time nodes gradually decreased, and the difference was statistically significant (*p* < 0.05), as is shown in Table 2. In different disease courses, the proportion of patients with an antibody titre ≥1:200^++^ accounted for the highest proportion, the proportion of patients with an antibody titre ≥1:100^++^ in the chronic phase was higher than that in the subacute phase and acute phase, and the proportion of patients with an antibody titre ≥1:200^++^ in the acute phase was higher than that in subacute phase and chronic phase. See Table 3 for details.

**Table 2:** Tube agglutination test results at each investigation time point (*n*, %).

	Baseline survey	First follow-up	Second follow-up	Third follow-up	*X* ^2^	*p*
≥1:100^++^	7 (7.00)	6 (7.30)	8 (13.79)	14 (43.75)	11.853	0.042
≥1:200^++^	89 (89.00)	76 (92.70)	50 (86.21)	18 (56.25)	121.251	<0.001
≥1:400^++^	4 (4.00)	0 (0)	0 (0)	0 (0)	12.121	0.007
Total	100 (100)	82 (100)	58 (100)	32 (100)		

Data in the table represent the number of cases (constituent ratio %); chi-square test was used to compare the results of tube agglutination test at each time point.

**Table 3:** Tube agglutination test results in different disease course (*n*, %).

	Acute phase (*n* = 100)	Subacute phase (*n* = 21)	Chronic phase (*n* = 11)	*X* ^2^	*p*
≥1:100^++^	7 (7.00)	9 (42.86)	5 (45.45)	24.510	<0.001
≥1:200^++^	89 (89.00)	12 (57.14)	6 (54.55)	12.235	0.002
≥1:400^++^	4 (4.00)	0 (0)	0 (0)	2.302	0.316
Total	100 (100)	21 (100)	11 (100)		

Data in the table represent the number of cases (constituent ratio); chi-square test was used to compare the results of tube agglutination test at each time point.

### 4.3. Liver function test results

All included patients underwent unified and standardised treatment, and liver function, renal function, and haematologic system tests were performed at each survey time point. Compared with the baseline survey, the proportion of patients with abnormal liver function test results increased at the first (89 patients, 89%) and second follow-up (94 patients, 94%) visits, and the proportion of patients with abnormal liver function test results at the third follow-up (27 patients, 27%) visit was much lower than that at the first two follow-up visits (*p* < 0.01); the trends of AST and TBIL were generally consistent (*p* < 0.01).

### 4.4. Renal function test results

Compared with the baseline investigation, the proportion of patients with abnormal renal function decreased to 13% at the third follow-up visit. Among them, the proportion of patients with elevated BUN increased at the first visit (22 patients, 22%) and the second visit (15 patients, 15%) (*p* < 0.01); those with elevated SCr began to decrease at the second visit (11 patients, 11%), falling to only 2 patients with elevated SCr at the third visit (*p* < 0.01).

### 4.5. Haematologic test results

Compared with the baseline survey, the proportion of those with haematologic abnormalities decreased at the first (60 patients, 60%) and second follow-up (49 patients, 49%) (*p* < 0.05) visits, but this number increased again at the third follow-up (69 patients, 69%) visit. The proportion of patients with abnormal RBC count decreased to 3% (*n* = 3) at the first follow-up visit and increased again (*p* < 0.05) at the third follow-up (*n* = 9, 9%) visit; the proportion of patients with elevated Hb decreased at the first (*n* = 31, 31%) and second follow-up (*n* = 24, 24%) visits and increased (*p* < 0.05) at the third follow-up (*n* = 34, 34%) visit. The proportion of patients with decreased PLT count began to decrease at the first follow-up visit (*n* = 5, 5%) (*p* < 0.05). The laboratory test results of patients with brucellosis at each survey time point are shown in Table 4.

**Table 4:** Laboratory test results of brucellosis patients at each investigation time point.

Exam	Specific indicators	Baseline survey (case)	First follow-up (case)	Second follow-up (case)	Third follow-up (case)	*X* ^2^	*p*
Liver function	ALT (0–40 U/L) increased	13	14	21	5	11.201	0.011
	AST (0–40 U/L) increased	11	27	23	11	13.821	0.003
	TBIL (3.4–20.5 umol/L) increased	24	48	50	11	48.605	<0.001
	Total	48	89	94	27	137.526	<0.001
Renal function	BUN (2.9–8.2 mmol/L) increased	4	22	15	11	12.866	0.005
	SCr (35–115 umol/L) Increased	29	22	11	2	31.696	<0.001
	Total	33	44	26	13	24.575	<0.001
Hematologic	RBC (3.5–5.5 × 1,012/L) increased	10	3	5	9	15.203	0.058
	WBC (4–10 × 10 ^9^/L) decreased	22	21	20	21	0.121	0.989
	Hb (110–160 g/L) increased	34	31	24	34	23.135	0.0371
	PLT (100–300 × 10 ^9^/L) decreased	15	5	0	5	20.267	<0.001
	Total	81	60	49	69	24.218	<0.001

ALT, alanine aminotransferase; AST, aspartate aminotransferase; TBIL, bilirubin; BUN, blood urea nitrogen; SCr, serum creatinine; RBC, red blood cell; WBC, white blood cell; Hb, hemoglobin; PLT, platelet; chi-square test was used to compare the laboratory test results of patients at each time point.

The biochemical parameter data at each survey time point were normal with equal variance, and the results of the sphericity test are shown in Table 5. Repeated measures ANOVA showed statistically significant differences in ALT, AST, TBIL, SCr and PLT levels at different time points (*p* < 0.05), as is shown in Table 6. Pairwise comparisons of biochemical indicator levels at the four time points showed that ALT levels were higher at both the first and second follow-up visits than at the third follow-up (*p* < 0.01) visit; in addition, AST levels were higher at the first and second follow-up visits than at the baseline survey and the third follow-up (*p* < 0.01) visit. The TBIL levels were increased at the first and second follow-up visits (*p* < 0.01) compared with the baseline survey, and the SCr levels decreased at the second follow-up visit compared with the first follow-up visit. Additionally, the SCr levels at the third follow-up visit were lower than that at the baseline survey and the first and second follow-up (*p* < 0.01) visits. The PLT levels were increased at the first and the second follow-up visit compared with the baseline survey but started to decrease at the third follow-up (*p* < 0.01) visit compared with the second follow-up visit. These results are shown in Table 7.

**Table 5:** Mauchly’s test of sphericity.

Within-subjects effect (time)	Mauchly’s *W*	Approximate chi-square	Degrees of freedom	*p*	Eplison		
					Greenhouse–Geisser	Hunyh–Feldt	Lower bound
ALT	0.137	32.250	5	0.051	0.390	0.392	0.333
AST	0.891	11.264	5	0.046	0.938	0.968	0.333
TBIL	0.010	443.390	5	0.037	0.361	0.362	0.334
BUN	0.113	567.038	5	0.044	0.402	0.404	0.333
SCr	0.290	120.811	5	0.043	0.599	0.610	0.333
RBC	0.013	540.521	5	0.005	0.369	0.370	0.332
WBC	0.003	538.018	5	0.001	0.368	0.370	0.333
Hb	0.665	39.866	5	0.047	0.808	0.830	0.335
PLT	0.850	15.924	5	0.007	0.918	0.947	0.433

ALT, alanine aminotransferase; AST, aspartate aminotransferase; TBIL, bilirubin; BUN, blood urea nitrogen; SCr, serum creatinine; RBC, red blood cell; WBC, white blood cell; PLT, platelet.

**Table 6:** Repeated biochemical measurements at each survey time point.

	Sum of squares of deviation from mean	Degrees of freedom	Mean square	*F*	*p*
ALT	8299.14	3	2766.38	2.014	0.042
AST	2219.42	2.813	789.12	3.370	0.022
TBIL	4035.34	1.084	1345.114	0.442	0.047
BUN	8536.99	1.206	2845.67	0.973	0.406
SCr	1101.497	1.798	3670.832	14.635	<0.001
RBC	232.04	1.107	209.561	1.010	0.325
WBC	220.457	1.105	199.45	10.30	0.321
Hb	329.648	2.425	135.916	0.186	0.869
PLT	455.437	2.755	165.981	7.973	<0.001

ALT, alanine aminotransferase; AST, aspartate aminotransferase; TBIL, bilirubin; BUN, blood urea nitrogen; SCr, serum creatinine; RBC, red blood cell; WBC, white blood cell; PLT, platelet.

**Table 7:** Results of pairwise comparison of biochemical indicators at each investigation time point.

		Baseline survey ( $\bar{X}\pm S$ , *n* = 100)	First follow-up ( $\bar{X}\pm S$ , *n* = 100)	Second follow-up ( $\bar{X}\pm S$ , *n* = 100)	Third follow-up ( $\bar{X}\pm S$ , *n* = 100)	*F*	*p*
Liver function	ALT	32.567 ± 64.430	28.777 ± 17.144	29.092 ± 20.154	20.073 ± 14.354	12.519	0.001
	AST	26.994 ± 16.277	32.159 ± 18.230	30.966 ± 17.453	26.807 ± 16.203	3.688	0.005
	TBIL	16.308 ± 11.630	22.815 ± 16.153	22.415 ± 14.920	24.977 ± 107.349	4.67	0.006
Renal function	BUN	8.173 ± 34.880	17.432 ± 102.147	6.547 ± 4.532	6.0425 ± 2.094	0.859	0.465
	SCr	83.906 ± 83.344	96.094 ± 45.914	77.167 ± 29.717	51.073 ± 26.633	25.145	0.001
Hematologic system	RBC	6.233 ± 16.421	4.817 ± 3.926	4.440 ± 0.679	4.378 ± 0.827	0.848	0.471
	WBC	5.061 ± 1.640	5.249 ± 1.539	5.232 ± 1.575	5.296 ± 3.014	0.421	0.738
	Hb	152.700 ± 33.081	150.96 ± 21.162	150.21 ± 23.415	151.42 ± 29.302	0.160	0.923
	PLT	154.885 ± 59.434	157.83 ± 50.074	184.16 ± 47.601	171.82 ± 49.867	7.513	0.001

ALT, alanine aminotransferase; AST, aspartate aminotransferase; TBIL, bilirubin; BUN, blood urea nitrogen; SCr, serum creatinine; RBC, red blood cell; WBC, white blood cell; PLT, platelet.

The ANOVA laboratory biochemical indicators showed that the ALT levels in the acute phase of brucellosis were lower than those in the subacute and chronic phases (*p* < 0.01). In addition, there were no significant differences in the AST, TBIL, BUN, SCr, RBC, WBC, Hb and PLT levels in the acute, subacute and chronic phases of brucellosis (*p* > 0.05). See Table 8.

**Table 8:** Laboratory biochemical parameters in patients with brucellosis in acute, subacute and chronic phases.

	Acute phase ( $\bar{X}\pm S$ , *n* = 100)	Subacute phase ( $\bar{X}\pm S$ , *n* = 21)	Chronic phase ( $\bar{X}\pm S$ , *n* = 11)	*F*	*p*
ALT	25.015 ± 13.094	16.175 ± 6.706	25.173 ± 9.877	4.52	<0.001
AST	25.687 ± 17.269	23.530 ± 7.147	29.491 ± 9.735	0.54	0.579
TBIL	16.526 ± 12.355	15.071 ± 8.871	13.240 ± 4.283	0.481	0.697
BUN	9.315 ± 39.984	6.408 ± 2.497	6.218 ± 1.907	0.084	0.933
SCr	88.201 ± 83.427	56.800 ± 20.172	66.455 ± 16.670	1.747	0.056
RBC	6.751 ± 18.823	4.603 ± 0.653	4.250 ± 0.901	0.228	0.701
WBC	5.000 ± 1.760	5.001 ± 1.297	4.780 ± 1.578	0.086	0.941
Hb	149.092 ± 32.599	157.950 ± 20.353	152.182 ± 30.558	0.675	0.552
PLT	155.125 ± 61.923	183.500 ± 40.841	147.455 ± 46.231	2.238	0.522

ALT, alanine aminotransferase; AST, aspartate aminotransferase; TBIL, bilirubin; BUN, blood urea nitrogen; SCr, serum creatinine; RBC, red blood cell; WBC, white blood cell; Hb, hemoglobin; PLT, platelet.

### 4.6. Clinical symptoms

At baseline, the main clinical manifestations of 100 patients with brucellosis were joint pain (81 cases, 81.0%), fatigue (55 cases, 55.0%) and fever (40 cases, 40.0%). Patients with joint pain mainly presented with pain in the knee joint (42 cases, 51.9%), shoulder joint (30 cases, 37.0%) and wrist joint (23 cases, 28.4%) and often presented with polyarticular discomfort (49 cases, 60.5%). At the first follow-up visit, joint pain (55 cases, 55.0%), muscle pain (27 cases, 27.0%) and fatigue (27 cases, 27.0%) were the main manifestations, and knee pain (24 cases, 43.6%) was more significant in patients with joint pain. In addition, compared with the baseline survey, those with fever (15 cases, 15.0%) and polyarticular pain (19 cases, 34.6%) were reduced (*p* < 0.01). At the second follow-up visit, joint pain (39 cases, 39.0%), fatigue (25 cases, 25.0%) and hyperhidrosis (13 cases, 13.0%) were predominant and knee pain (13 cases, 33.3%) was more significant in patients with joint pain; those with fever symptoms (9 cases, 9.0%) and polyarticular pain (9 cases, 9.0%) were reduced compared with those at the baseline survey (*p* < 0.01). Joint pain (19 patients, 19.0%) was the most predominant manifestation at the third follow-up visit and was most prominent in patients with shoulder pain (6 patients, 31.6%); there were fewer patients with fever (2 patients, 2.0%), polyarticular pain (3 patients, 15.8%) and muscle pain (2 patients, 2.0%) (*p* < 0.01) compared with the baseline survey (see Table 9).

**Table 9:** Clinical symptoms of brucellosis patients at each investigation time point.

	Baseline survey	First follow-up	Second follow-up	Third follow-up	*X*^2^/*Z*	*p*
	(e.g., *n* = 100)	(e.g., *n* = 100)	(e.g., *n* = 100)	(e.g., *n* = 100)		
Fever (>37.2°C)	40	15	9	2	59.590	<0.001
Asthenia	55	27	25	6	60.325	<0.001
Hyperhidrosis	38	9	13	1	59.171	<0.001
Arthralgia	81	55	39	19	29.688	<0.001
Sacrum	15	10	5	3	11.461	0.009
Ilium	7	1	4	3	5.195	0.158
Shoulder	30	8	8	6	34.306	<0.001
Genu	42	24	13	4	48.816	<0.001
Elbow	20	1	2	0	50.329	<0.001
Wrist	23	13	4	1	32.040	<0.001
Ankle	15	12	6	2	12.869	0.005
Spine	1	0	0	0	3.008	0.390
Monoarticular	25	31	24	14	8.288	0.04
Polyarticular	49	19	9	3	78.250	<0.001
Migratory	7	5	6	2	2.947	0.400
Muscular pain	37	27	13	2	43.877	<0.001
Chills	6	4	2	2	3.257	0.354
Cold intolerance	9	6	5	1	6.584	0.086
Cough	12	10	2	0	18.440	0.004
Expectoration	6	2	1	0	9.891	0.02
Sleep disorder	8	0	2	1	14.489	0.002

Chi-square test or Fisher exact test was used to compare frequency of clinical symptoms at each time point.

Joint pain (81 cases, 81.0%), fatigue (55 cases, 55.0%) and fever (40 cases, 40.0%) were the main clinical symptoms in the acute stage. The clinical symptoms of patients with different courses of the disease were analysed. The proportion of patients with hyperhidrosis, chills, fear of cold, cough and expectoration in the acute phase (38.0%, 2.0%, 7.0%, 12.0% and 6.0%) was higher than that in subacute and chronic phases (*p* < 0.01). Compared with patients with acute fever, the proportion of patients with subacute fever (20 patients, 64.5%) increased, and the proportion of patients with joint pain decreased (2 patients, 9.5%). Patients with fever in the chronic phase (1 case, 9.1%) were lower than those in the acute and subacute phases (*p* < 0.01); the proportions of patients with fatigue (3 cases, 27.3%), joint pain (4 cases, 36.4%) and muscle pain (1 case, 9.1%) in the chronic phase were lower than in the acute phase (55.0%, 81.0% and 37.0%, respectively) and higher than in the subacute phase (9.5%, 9.5% and 0.0%, respectively) (*p* < 0.01). See Table 10.

**Table 10:** Clinical symptoms of brucellosis patients with different course.

	Acute phase	Subacute phase	Chronic phase	*X*^2^/*Z*	*p*
	(*n* = 100)	(*n* = 21)	(*n* = 11)		
Fever (>37.2°C)	40	20	1	17.851	<0.001
Asthenia	55	2	3	23.955	0.001
Hyperhidrosis	38	0	0	17.076	0.002
Arthralgia	81	2	4	44.123	<0.001
Sacrum	15	0	0	5.415	0.067
Ilium	7	0	0	4.978	0.083
Shoulder	30	2	3	4.165	0.125
Genu	42	0	1	16.952	0.002
Elbow	20	0	0	12.206	0.002
Wrist	23	0	0	8.913	0.012
Ankle	15	0	0	33.730	<0.001
Spine	1	0	0	0.322	0.851
Monoarticular	25	2	2	2.525	0.283
Polyarticular	49	0	0	24.937	<0.001
Migratory	7	0	1	1.687	0.43
Muscular pain	37	0	1	13.861	0.001
Chills	2	0	0	12.361	0.002
Cold intolerance	7	0	0	14.533	0.001
Cough	12	0	0	16.810	0.002
Expectoration	6	0	0	14.091	0.001
Sleep disorder	8	0	0	14.980	0.001

Chi-square test or Fisher exact test was used to compare frequency of clinical symptoms at each time point.

### 4.7. Factors affecting the prognosis of patients with brucellosis

#### 4.7.1. Univariate logistic regression analysis

Of the 100 patients, 68 were cured and 32 were uncured at the third follow-up visit. Univariate logistic regression was used to analyse gender, age, clinical symptoms and laboratory test results; the results are shown in Table 11. Gender was not a factor influencing poor prognostic outcome (not cured) [*p* = 0.986, 95% confidence ratio (CI) = 0.311–3.281], and patients aged ≥45 years had a higher risk of a poor prognostic outcome (not cured) than patients younger than 45 years old [*p* = 0.021, odds ratio (OR) = 6.336, 95% CI = 1.318–30.458]. Comparing the laboratory test results between the two groups, patients with elevated SCr (*p* = 0.015, OR = 1.076, 95% CI = 0.010–0.062) and patients with elevated Hb had a higher risk of a poor prognostic outcome (not cured) (*p* = 0.009, OR = 4.918, 95% CI = 1.484–16.295). In addition, there was no statistically significant difference in the proportion of fatigue, shoulder and knee pain, type of joint pain, whether the medication was taken on time, and abnormal ALT, AST, RBC, WBC, and PLT results between the two groups (*p* > 0.05).

**Table 11:** Univariate logistic regression analysis of adverse prognostic outcomes in brucellosis patients.

Independent variable	Cured (*n* = 68)	Not cured (*n* = 32)	*p*	OR	95% CI
Female	31 (47.69)	11 (31.43)	0.986	1.011	0.311–3.281
Age ≥45 years	48 (73.85)	32 (91.43)	0.021	6.336	1.318–30.458
Asthenia	3 (4.62)	3 (8.57)	0.894	1.215	0.070–21.226
Arthralgia	15 (23.08)	4 (11.43)	1.000	3.541	2.156–7.312
Shoulder pain	4 (6.15)	2 (5.71)	0.620	2.213	0.096–51.255
Knee pain	3 (4.62)	1 (2.86)	0.429	4.784	0.99–232.175
**Type of joint pain**					
Monoarticular pain	12 (18.46)	2 (5.71)	1.000	4.213	1.662–9.583
Polyarticular pain	2 (3.08)	0 (0)	0.999	1.324	0.547–4.783
Joint migratory pain	1 (1.54)	1 (2.86)	1.000	1.631	1.242–7.894
On-time medication	9 (13.85)	1 (2.86)	0.395	2.058	0.390–10.872
ALT increased	5 (7.70)	1 (2.86)	0.831	0.818	0.130–5.150
AST increased	8 (12.31)	3 (8.57)	0.598	0.494	0.036–6.785
TBIL increased	14 (20.59)	4 (12.50)	0.348	0.548	0.156–1.924
BUN increased	10 (14.71)	7 (21.88)	0.586	1.396	0.420–4.635
SCr increased	19 (32.76)	1 (2.86)	0.015	1.076	0.010–0.062
RBC increased	9 (13.85)	5 (14.29)	0.892	1.120	0.218–5.759
WBC decreased	13 (20.00)	10 (28.57)	0.059	3.501	0.955–12.829
Hb increased	18 (27.69)	16 (45.71)	0.009	4.918	1.484–16.295
PLT decreased	3(4.62)	32(91.43)	0.977	0.969	0.111–8.466

Data in the table represent the number of cases (proportion); ALT, alanine aminotransferase; AST, aspartate aminotransferase; TBIL, bilirubin; BUN, blood urea nitrogen; SCr, serum creatinine; RBC, red blood cell; WBC, white blood cell; Hb, hemoglobin; PLT, platelet.

#### 4.7.2. Multivariate logistic analysis

Age, elevated SCr and elevated Hb were known to be risk factors for a poor prognosis in patients with brucellosis by univariate logistic analysis, and these three variables and risk factors that may trigger chronicity in patients were subsequently analysed by multivariate logistic analysis. The results are shown in Table 12. The results showed that joint pain (*p* = 0.001, OR = 3.652, 95% CI = 1.379–9.672), single joint pain (*p* = 0.001, OR = 6.356, 95% CI = 4.660–8.669), elevated SCr (*p* = 0.017, OR = 15.804, 95% CI = 1.644–151.966) and elevated Hb (*p* = 0.014, OR = 1.219, 95% CI = 0.065–1.736) were risk factors for uncured patients. The other variables included in the multivariate logistic analysis showed no significant difference between the two groups (*p* > 0.05).

**Table 12:** Multivariate logistic regression analysis of adverse prognostic outcomes in patients with brucellosis.

Variable	*p*	OR	95% CI
Female	0.986	1.011	0.311–3.281
Age ≥45 years	0.144	0.234	0.036–1.626
Asthenia	0.833	0.806	0.045–14.324
Arthralgia	0.001	3.652	1.379–9.672
Shoulder pain	0.324	0.172	0.005–5.658
Knee pain	0.429	0.193	0.003–11.363
Monoarticular pain	0.001	6.356	4.660–8.669
Polyarticular pain	0.997	9.522	6.730–11.641
Joint migratory pain	0.661	4.856	3.611–6.713
On-time medication	0.434	0.517	0.099–2.706
ALT increased	0.876	1.224	0.097–15.390
AST increased	0.951	1.060	0.167–6.714
TBIL increased	0.149	3.111	0.665–14.554
BUN increased	0.404	0.500	0.098–2.542
SCr increased	0.017	15.804	1.644–151.966
RBC increased	0.967	0.966	0.188–4.958
WBC decreased	0.077	0.305	0.082–1.135
Hb increased	0.014	1.219	0.065–1.736
PLT decreased	0.992	1.011	0.118–8.691

ALT, alanine aminotransferase; AST, aspartate aminotransferase; TBIL, bilirubin; BUN, blood urea nitrogen; SCr, serum creatinine; RBC, red blood cell; WBC, white blood cell; Hb, hemoglobin; PLT, platelet.

## 5. Discussion

Brucellosis is a widespread global zoonosis disease and has been found in humans and animals in more than 170 countries (4, 13); it is primarily concentrated in countries in the Middle East, along the Mediterranean coast, and in sub-Saharan Africa, Asia, and South America (14–16). In many provinces (municipalities directly under the central government and autonomous regions) in China, there are varying degrees of occurrence and prevalence of brucellosis. The most serious issue occurred in the 1950s and 1960s, with a steady decline in the 1970s and 1980s. However, cases showed a rising trend in the middle and late 1990s, resulting in an epidemic situation going into the 21st century. At present, brucellosis in China is mainly distributed in provinces and regions engaged in the animal husbandry and breeding industry in southwest and northwest China, and the incidence has been stable at about 20/100,000 for nearly five consecutive years (17).

Inner Mongolia is a historical epidemic area of brucellosis. In the 1980s, the epidemic situation was effectively controlled. However, since the late 1990s, especially into the 2000s, the epidemic has rebounded rapidly because of the vigorous development of animal husbandry. In 2017, there was a steep rise with 7,744 new cases reported in the region. In 2018, 10,111 cases were reported, and the number continued to rise from there: 14,148 in 2019, 17,478 in 2020, and 21,910 in 2021. In 2021, the number of cases in Inner Mongolia accounted for 51.32% of the total number of cases reported in China, ranking first in the country. This was also a record high for reported cases in the region. In fact, since 2017, the number of reported cases in Inner Mongolia has ranked first in China for five consecutive years, and the annual growth rate of the number of cases from 2017 to 2021 was 29.49%. This has seriously affected the health and economic development of the people in the region and has become a major public health issue (18, 19).

In this study, 100 patients with acute brucellosis were followed up with for 6 months. The differences in laboratory test results over the different disease stages were repeatedly measured, and the changes in serum and biochemical parameters during this time were analysed. This study also explored the risk factors affecting the prognosis of patients with brucellosis.

A total of 100 patients with brucellosis were included, and the animals these patients were exposed to were mainly cattle, sheep, pigs, and dogs. In addition, 98.98% were exposed to two or more animals at the same time. The patients had an average age of 53.810 ± 0.993 years, and the age of onset and gender characteristics of brucellosis were consistent with previous studies (20, 21). In this study, the average age of disease onset was 53.8 years, which may be related to the fact that rural residents over 50 years of age choose to work in agriculture at home and raise livestock during farming leisure time. In addition, elderly adults have less physical strength than younger adults, and brucellosis awareness was lower in the elderly than in the young population (22). We similarly observed gender differences in this study, including a higher proportion of male patients than females.

In the SAT test, 100 patients in the acute phase were treated by standardised unified therapy. At the first follow-up, 4 patients had SAT agglutination antibody titers ≥1:400^++^, and 89 patients had SAT agglutination antibody titers ≥1:200^++^; at the third follow-up, patients with no antibody titers ≥1:400^++^, and those with antibody titers ≥1:200^++^ decreased significantly. García Casallas et al. (23) stated that brucellosis can develop and persist as a chronic disease, becoming a granulomatous disease capable of affecting any organ system. It is evident that the early diagnosis and standardised treatment of patients with brucellosis in the acute phase is essential.

Brucellosis is a systemic infectious disease, which often involves a variety of organs and systems, causing multiple organ-destructive diseases in patients, often resulting in complications. Both ALT and AST are involved in various physiological and biochemical metabolic reactions in the body; the anaerobic glycolysis of glucose leads to the increase of transaminases, so they are commonly used to reflect liver function, and their ratio can predict a variety of tumours and their prognoses (24, 25). Compared with the baseline investigation, there were significantly more patients with abnormal liver function levels and increased AST and TBIL levels during the first and second follow-up visits. This may be related to the fact that the patients with brucellosis were in the acute progression of brucellosis at this time (26–28). With treatment, the proportion of patients with abnormal liver function test results gradually decreased, decreasing to 27% at the third follow-up visit. Compared with the results at the first and second follow-up visits, the ALT and AST levels decreased. These results are consistent with the results of Hosseini SM et al. (29), reporting an increase of 26% in AIT enzyme and 25% in AST enzyme in rats after infection. Another study (30) also reported increased levels of liver enzymes in rats with chronic brucellosis and stage. The Kazak et al. (31) study had similar findings: patients presented with abnormally high AST and ALT levels, noting that the rate of liver involvement in brucellosis was high and that high levels of AST and ALT cannot be ignored. The positive rate of SAT and the antibody titre in 100 patients with acute brucellosis decreased gradually with the course of the disease, and the ALT level in patients with subacute brucellosis was lower than that in patients with acute and chronic brucellosis. There was a certain trajectory in the changes between the two, which provided a theoretical basis for finding markers associated with chronic brucellosis.

Brucellosis is preventable and treatable. In the 100 patients with acute brucellosis included in this study, the main clinical manifestations were joint pain (81%), fatigue (55%), and fever (40%) at the baseline survey, which was consistent with what was noted in a systematic evaluation study by Zheng et al. (32) After receiving unified standardised treatment, the relevant symptoms were gradually reduced or eliminated, and patients whose symptoms manifested as fever, fatigue, hyperhidrosis, and polyarticular pain had been reduced at the first follow-up visit. The proportion of patients with joint pain decreased at the third follow-up visit, and the number of patients complaining of shoulder joint and elbow joint pain decreased at the first follow-up visit. Therefore, the relevant clinical symptoms of the patients in the acute phase of brucellosis can be effectively relieved and eliminated through a full course of standardised treatment.

The results of this study showed that there were significant differences in the AST, ALT and PLT results at the baseline survey and subsequent follow-up visits.

Under the influence of *Brucella*, chronic body consumption and hypersplenism often accompany an abnormal haemogram (33, 34). Creatinine is a measure of glomerular filtration function and the risk of chronicity increases when SCr increases. An abnormal haemogram is generally a transient complication, which is easily converted after standardised treatment. Haemoglobin is a protein that transports oxygen in RBCs, and this study’s results show that when Hb is elevated in patients with brucellosis and not treated timely, the risk it of chronicity increases. In the present study, there were no significant differences in the RBC, WBC, Hb and PLT levels in the acute, subacute, and chronic phases of brucellosis (*p* > 0.05), which is in agreement with the literature (35). However, in Hosseini’s et al. (29) study, the number of WBCs was increased by 21% in rats during the acute phase of the infection. The difference between results could be due to the different study subjects. The chronicity rate of brucellosis in this study was 11%, and the logistic regression analysis of the risk factors affecting the prognosis of brucellosis revealed that an age ≥45 years, joint pain, and single joint pain were risk factors for patients who were not cured. In addition, some patients had elevated SCr and Hb levels, and if left untreated, elevated SCr and Hb were also risk factors for patients who were not cured. The results of the multivariate logistic regression analysis showed that joint pain, single joint pain and elevated SCr and Hb were all risk factors for adverse outcomes. When other factors remained unchanged, the probability of chronicity increased by a factor of 3.652 (*p* < 0.05) in patients with arthralgia symptoms and by a factor of 6.356 (*p* < 0.05) in patients presenting with single joint pain. If patients with elevated SCr and Hb were not monitored and treated timely, the risk of chronicity increased by a factor of 15.804 (*p* < 0.05) in patients with elevated SCr and by a factor of 1.219 (*p* < 0.05) in patients with elevated Hb. Therefore, according to the risk factors, it is recommended that the treatment cycle should be extended for such patients.

This study had some limitations. First, this study only investigated the risk factors affecting the prognosis of brucellosis; it cannot be concluded that there is any causal association. Second, the sample source of this study is relatively single-centre, and the conclusions drawn may only be regional. The next step is to expand the size of the study and establish a cohort for a long-term, in-depth study.

## 6. Conclusion

The trajectory of changes in patient SAT posirates and antibody titers can be used to distinguish patients with chronic brucellosis. Brucellosis is preventable and treatable, and a standardised treatment can be effective in reducing the clinical symptoms of patients. If patients are not treated in a timely manner, joint pain, monoarticular pain, and elevated SCr are risk factors for patients who are not cured. Therefore, the treatment cycle for these patients should be extended.

## Data availability statement

The raw data supporting the conclusions of this article will be made available by the authors, without undue reservation.

## Ethics statement

The studies involving humans were approved by the Ethics Committee of the Center for Disease Control and 463 Prevention of Inner Mongolia Autonomous Region (Approval No.: 2022011601). The studies were conducted in accordance with the local legislation and institutional requirements. The participants provided their written informed consent to participate in this study. Written informed consent was obtained from the individual(s) for the publication of any potentially identifiable images or data included in this article.

## Author contributions

YW and HB designed the study. LB, NT, AZ, HM, XL, and BW performed clinical examinations and collected patient data. LB and NT drafted the manuscript. All authors read and approved the submitted version of the manuscript.
